# Omega-3 Supplementation and Heart Disease: A Population-Based Diet by Gene Analysis of Clinical Trial Outcomes

**DOI:** 10.3390/nu13072154

**Published:** 2021-06-23

**Authors:** Maria Luz Fernandez, Sarah A. Blomquist, Brian Hallmark, Floyd H. Chilton

**Affiliations:** 1 Department of Nutritional Sciences, University of Connecticut1, Storrs, CT 06268, USA; 2 Department of Nutritional Sciences, The University of Arizona, Tucson, AZ 85721, USA; sblomquist@email.arizona.edu (S.A.B.); Floyd.chilton@arizona.edu (F.H.C.); 3 BIO5 Institute, The University of Arizona, Tucson, AZ 85721, USA; bhallmark@statlab.bio5.org

**Keywords:** *n*-3 polyunsaturated fatty acids, genetic variation, *FADS* gene, coronary heart disease, precision nutrition

## Abstract

Omega-3 (*n*-3) polyunsaturated fatty acids (PUFA) and their metabolites have long been recognized to protect against inflammation-related diseases including heart disease. Recent reports present conflicting evidence on the effects of *n*-3 PUFAs on major cardiovascular events including death. While some studies document that *n*-3 PUFA supplementation reduces the risk for heart disease, others report no beneficial effects on heart disease composite primary outcomes. Much of this heterogeneity may be related to the genetic variation in different individuals/populations that alters their capacity to synthesize biologically active *n*-3 and omega 6 (*n*-6) PUFAs and metabolites from their 18 carbon dietary precursors, linoleic acid (LA, 18:2 *n*-6) and alpha-linolenic (ALA, 18:3, *n*-3). Here, we discuss the role of a *FADS* gene-by-dietary PUFA interaction model that takes into consideration dietary exposure, including the intake of LA and ALA, *n*-3 PUFAs, eicosapentaenoic acid (EPA) and docosahexaenoic acid (DHA) in determining the efficacy of *n*-3 PUFA supplementation. We also review recent clinical trials with *n*-3 PUFA supplementation and coronary heart disease in the context of what is known about fatty acid desaturase (*FADS)* gene-by-dietary PUFA interactions. Given the dramatic differences in the frequencies of *FADS* variants that impact the efficiency of *n*-3 and *n*-6 PUFA biosynthesis, and their downstream signaling products among global and admixture populations, we conclude that large clinical trials utilizing “one size fits all” *n*-3 PUFA supplementation approaches are unlikely to show effectiveness. However, evidence discussed in this review suggests that *n*-3 PUFA supplementation may represent an important opportunity where precision interventions can be focused on those populations that will benefit the most from *n*-3 PUFA supplementation.

## 1. Introduction

Lipid signaling metabolites including eicosanoids, resolvins, protectins, lipoxins and endocannabinoids all play critical roles in physiology and pathophysiology. These metabolites are formed from long-chain polyunsaturated fatty acids (LC-PUFAs, ≥20 carbons-PUFAs) belonging to the *n*-6 (adrenic acid (ADA; 22:4, *n*-6) and arachidonic acid (ARA; 20:4, *n*-6)) and *n*-3 (docosapentaenoic acid (DPA; 22:5, *n*-3), eicosapentaenoic acid (EPA; 20:5, *n*-3) and docosahexaenoic (DHA; 22:6, *n*-3)) families. Humans can synthesize LC-PUFA from their essential 18 carbon (18C) precursors, linoleic acid (LA; 18:2, *n*-6) or alpha-linolenic acid (ALA; 18:3, *n*-3) or obtain them directly from dietary sources [1]. In addition to serving as precursors for signaling molecules, LC-PUFAs linked to complex lipids such as phospholipids, lysophospholipids and cholesterol esters have important structural and functional roles [2]. *N*-3 and *n*-6 LC-PUFAs and their bioactive metabolites typically have opposing effects on inflammation and coagulation with ARA and its oxylipin metabolites, including eicosanoids such as prostaglandins, thromboxanes and leukotrienes associated with pro-inflammatory and pro-coagulant properties [3,4], while *n*-3 LC-PUFAs (EPA and DHA) and their metabolites have anti-inflammatory and pro-resolving activities [5]. 

Dietary patterns have significantly deviated from ancestral diets and a clear example of this are the dietary levels and ratios of 18C-PUFAs and LC-PUFAs [6]. The vast majority of ingested PUFAs are the *n*-6 and *n*-3 18C-PUFAs, LA and ALA, respectively [1]. The increased consumption of *n*-6 PUFAs largely driven by the commercial refining of LA-rich seed oils in the 20th century induced a dramatic increase in levels of total PUFAs and particularly LA, thereby dramatically shifting the balance of ALA to LA [1]. These alterations in turn lead to an imbalance between *n*-6 pro-inflammatory and *n*-3 anti-inflammatory (or pro-resolving) mediators. These changes have been proposed to play a role in the high-risk inflammatory diseases observed in Western civilization [5,7]. 

Given the effects of *n*-3 LC-PUFAs on hyperlipidemia, brain development/disorders and their capacity to be converted to *n*-3 anti-inflammatory (or pro-resolving) mediators, altering dietary levels of *n*-3 LC-PUFAs has been a nutritional strategy to improve multiple disease conditions [8]. Accordingly, numerous clinical interventions as well as epidemiological analysis have reported protective effects of *n*-3 LC-PUFAs with cardiovascular disease (CVD) [9,10], inflammation [11], Alzheimer’s disease [12], depressive symptoms and bipolar disorder [13], cancer [14,15] and autoimmune diseases [16]. Additionally, higher concentrations of *n*-3 LC-PUFAs in erythrocytes are associated with decreased risk of cardiovascular deaths [17,18], and schizophrenic patients have lower levels of *n*-3 LC-PUFAs than healthy controls [19,20]. More recently, it has been shown that disease severity is inversely related to concentrations of circulating *n*-3 LC-PUFAs in COVID-19 patients [21].

However, several recent clinical trials [22,23] and meta-analyses [24,25] have questioned the efficacy of *n*-3 LC-PUFA in CVD. Conversely, other recent clinical trials [26,27], as well as an analysis of recent clinical studies suggest that *n*-3 LC-PUFAs do provide protective effects against secondary cardiovascular outcomes [28]. An extensive systematic assessment (79 RCTs, 112,059 participants) suggests that increasing *n*-3 LC-PUFAs, EPA and DHA has little or no effect on mortality or cardiovascular health (evidence mainly from supplement trials) [25]. However, they do lower plasma triglycerides and increase HDL and may lower myocardial infarction, arrythmia and heart failure [25]. Clearly, a major obstacle in this field is determining the biological source of heterogeneity leading to such disparate results. This will be necessary for more individualized dietary *n*-3 LC-PUFAs recommendations and to design clinical trials with *n*-3 LC-PUFAs in different populations.

In addition to dietary PUFA and LC-PUFA intake, genetic variations in LC-PUFA biosynthesis is a key determinant of the levels of LC-PUFAs, their lipid intermediates and their bioactive metabolites. Numerous candidate genes and GWAS studies over the past decade (reviewed in [29]) show that the efficiency of LC-PUFA biosynthesis is determined by variations in the genes that encode the fatty acid desaturase (*FADS*) and fatty acid elongase (*ELOVL*) enzymes. Patterns of genetic variations, particularly within the *FADS* gene cluster, are closely associated with ancestry, clearly demonstrating that the capacity to synthesize LC-PUFAs s is not uniform across all human populations.

The objective of this review is to analyze the findings of the effects of *n*-3 LC-PUFAs on cardiovascular events and highlight where diet-by-gene interactions that alter the endogenous synthesis of *n*-6 or *n*-3 LC-PUFAs may be a major confounder in clinical trial results. Given the marked ancestry-based differences in *n*-6 or *n*-3 LC-PUFAs biosynthesis, this review also points out that large clinical trials with diverse study populations have likely obscured the benefits *n*-3 LC-PUFAs that might be revealed by better focused interventions in specific populations. 

## 2. Alterations in Dietary Fatty Acids and Health Consequences

Chronic health problems in the US including obesity [30], cardiovascular disease [31], hypertension [32] and type-2 diabetes (T2D) [33] can be partially attributed to diet. The modern Western diet (MWD) evolved due to advancing technology after the Industrial Revolution, drastically changing the diet of our ancestors by adding food and food types that were previously unavailable. These foods include dairy products, cereal grains, refined sugars, and refined vegetable oils (high in *n*-6 fatty acids) [5]. The trend was exacerbated when meat from grain fed cattle became the norm in the US over the past 100 years [34]. The MWD has been associated with many of the current health problems related to chronic disease as well as the increase in health expenses in the US [35]. Several organizations including the United States Department of Agriculture (USDA) [36], the American Heart Association [37] and the American Cancer Society [38] have released dietary guidelines to address these issues. However, blanket recommendations do not consider individual variability in the response to diet, especially those that may be due to genetic variation or ancestry. Precision nutrition, or dietary recommendations based on each individual genetic makeup, will continue to become more important for the health of diverse populations [39]. 

The intake of *n*-6 PUFAs dramatically increased in the last 60 years in the US largely as a result of recommendations to lower saturated fat in order to decrease plasma LDL cholesterol [37]. The lowering of LDL cholesterol was once thought to be the most important factor in reducing the risk for heart disease [40]. However, while hypercholesterolemia is undoubtedly a key factor in the development of atherosclerosis, heart disease is very complex and other metabolic dysregulations including hypertriglyceridemia, low grade inflammation and oxidative stress also significantly contribute to CVD risk, myocardial infarction and cardiac death [41,42]. 

The substitution of dietary saturated fat with largely *n*-6 PUFAs markedly enhanced circulating levels of *n*-6 PUFAs, *n*-6 to *n*-3 PUFA ratios, and lowered circulating *n*-3 LC-PUFA concentrations [1]. Notably, contemporary diets such as the MWD typically have *n*-6 to *n*-3 PUFA ratios > 10:1 and as high as 20:1 [43]. Available data based on knowledge of food supply indicates dietary *n*-6 PUFA levels were 3- to 4-fold lower before the late 19th and early 20th century [44]. Other current dietary practices such as plant-based diets have also been associated with changes in PUFA and LC-PUFA levels and particularly a deficiency in *n*-3 LC-PUFAs [45]. There has been a robust debate among scientists about the potential risks of the rapid increase of LA in contemporary diets and several studies have warned against disease outcomes that could result from these rapid changes [46,47,48]. Given the shared enzymatic steps involved in the processing of LA and ALA, and the limited capacity of the pathway, these dietary PUFAs and their metabolic intermediates compete in the liver and other tissues for the desaturation and elongation reactions that produce *n*-6 and *n*-3 LC-PUFAs. A marked increase in LA along with competition between *n*-6 and *n*-3 substrates within the pathway has been shown in animal and human models to shift the pathway toward the biosynthesis of high levels of *n*-6 LC-PUFA and decreased levels of *n*-3 LC-PUFAs [48,49,50]. This shift in the balance of *n*-6 to *n*-3 LC-PUFAs in turn impacts the metabolic products and their ratios (such as *n*-6 to *n*-3 oxylipins and endocannabinoids), potentially impacting systemic inflammation and the risk for multiple chronic diseases. 

## 3. Genetic Variation in LC-PUFA Biosynthesis

Until two decades ago, the capacity of humans to synthesize *n*-6 to *n*-3 LC-PUFAs from dietary PUFAs was thought to be limited (3–4% of energy) and uniform in all humans. However, numerous recent studies and reviews point out that genetic variation, particularly in the *FADS* gene cluster, alters the biosynthetic efficiency of LC-PUFA biosynthesis [7,29,51]. Perhaps most surprising is the striking differences in the frequency of *FADS* genetic variants in different ancestry populations [52,53]. This was initially discovered when comparing the *FADS* genotypic frequencies and haplotype patterns in African versus European ancestry populations [51,52,53]. The *FADS* haplotype structure is defined by two major linkage disequilibrium (LD) blocks. A set of 28 SNPs in the primary haplotype block most clearly distinguishes the “ancestral” and “derived” haplotypes. These haplotypes largely account for the associations observed in circulating and tissue LC-PUFA levels [54]. We and others initially demonstrated that African ancestry populations have much higher frequencies (versus European or Asian ancestry populations) of *FADS*-derived haplotype variants associated with enhanced conversion of dietary LA to ARA and of ALA to EPA and DHA [51,52,55]. We recently published the *FADS* ancestral and derived haplotype proportions in 42 modern human populations from around the globe, again demonstrating that African and Amerind populations have the highest proportions of the derived and ancestral haplotypes, respectively [52]. Modern European and Asian populations had both the derived and ancestral haplotype in different proportions [52]. Approximately 80% of African Americans carry two copies of the derived haplotype *FADS* variants (such as GG at rs174537) compared to only ∼45% of European Americans. This, taken together with the fact that *n*-6 LA represents 6–8% of daily energy (with *n*-6 to *n*-3 PUFA ratios >10:1) in contemporary diets, predicts that individuals with both copies of the derived haplotype *FADS* variants will synthesize significantly higher levels of ARA. This is observed when comparing ARA levels in African American versus European American populations [51,55].

**Figure 1:**
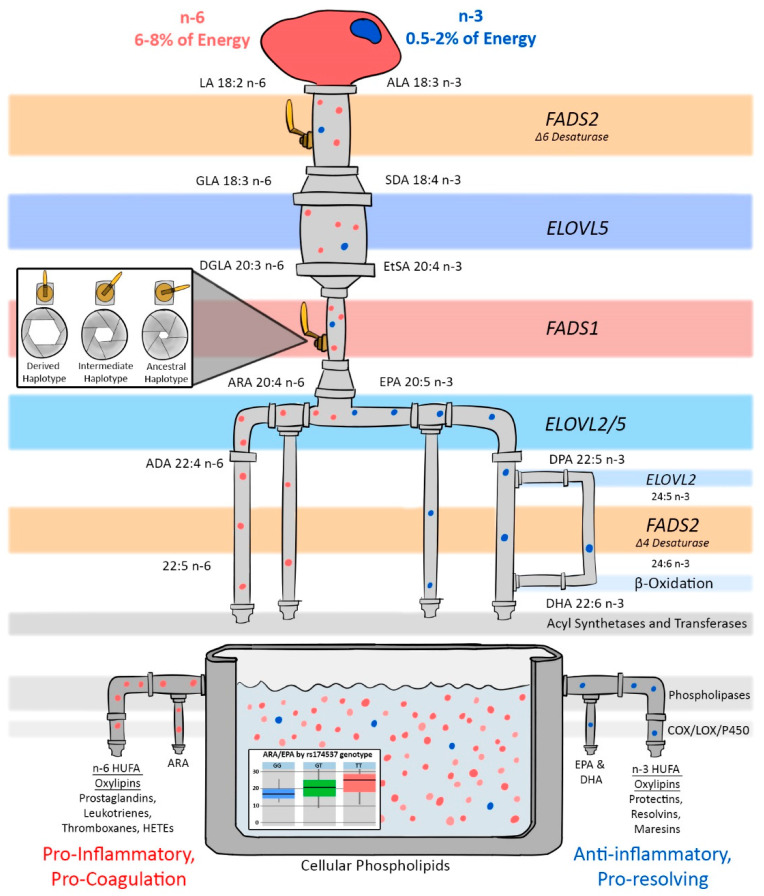
Illustration of the LC-PUFA biosynthesis pathway showing how *FADS* haplotypes utilize dietary *n*-3 and *n*-6 PUFAs for the biosynthesis of LC-PUFAs and their bioactive metabolites such as prostaglandins, oxylipins, leukotrienes, thromboxanes, HETEs, protectins and resolvins. Recent studies suggest that genetic variation in *FADS1* plays a critical role in impacting the efficiency of the FADS1 (∆5 desaturase) biosynthetic step and this represents the rate-limiting step in the formation of LC-PUFA-containing structural lipids and signaling molecules [57,59]. In this illustration, genetic control of the FADS1 and FADS2 steps are represented by the valve handle in the FADS steps. When the handle is wide open, as is the case for people with two copies of the derived haplotype, individuals have the ability to more efficiently synthesize LC-PUFAs from dietary 18C-PUFA precursors *(n*-3 and *n*-6). When the handle is partially closed, as is the case for people with one copy, individuals have intermediate efficiency. The valve handle is almost closed for people with an ancestral haplotype, and thus the LC-PUFA biosynthesis efficiency is lower. Additionally, the efficiency through the pathway has a direct role on the ratio of *n*-3 and *n*-6 LC-PUFAs that accumulate in phospholipid pools (as indicated in the container in this figure). FADS = fatty acid desaturase; ELOVL = fatty acid elongase; LA = linoleic acid; ALA= alpha linolenic acid; DGA = gamma linolenic acid; DGLA= dihomo-gamma-linoleic acid: EsTA= eicosotraenoic acid; EPA=Eicosopentanoic acid; DHA = Docosahexanoic acid; ARA= araquidonic acid; DPA = Docosopentanoic acid; COX = Cyclooxygenase; LOX= lipoxygenase and HUFA-highly unsaturated fatty acids.

In contrast, the ancestral haplotype associated with a more limited capacity to synthesize LC-PUFAs is nearly fixed in Native American populations, and we have recently demonstrated that high Amerind (genetically related to the Indigenous peoples of the Americas) ancestry Hispanic populations (largely from Mexico) also have high frequencies the ancestral haplotype (40–50% versus ~11% of European Americans) [56]. Recent studies have provided evidence that pathway efficiency, especially at the FADS1 desaturation step (Figure 1), is lower in individuals with the ancestral haplotype [57,58]. In 1997, Okuyama and colleagues [59] made a compelling case that excess LA plus the dramatic increase in the dietary LA to ALA ratio leads to *n*-3 LC-PUFA deficiency in certain populations. This ‘Omega-3 Deficiency Syndrome’ has been proposed to increase the risk of western-type cancers, cardiovascular and cerebrovascular diseases because the levels of *n*-3 LC-PUFAs and their metabolites play critical roles in innate immunity, energy, cardiometabolic health and brain development and function [57]. In addition, *n*-6 and *n*-3 LC-PUFAs and their metabolic products generally have opposing effects on immunity and inflammation [3,4,5]. 

Figure 1 illustrates the potential effects of high dietary levels of LA and LA to ALA ratios >10:1 on the levels of *n*-6 and *n*-3 LC-PUFAs, especially at the rate-limiting FADS1 biochemical step. Kothapalli et al. showed that the metabolic flux through the FADS1 step, as measured by the product to precursor ratio of ARA to dihomogammalinolenic acid (DGLA), increases by 84% when examining the differences between the DD to II genotype of the *FADS* insertion–deletion (Indel), rs66698963 [60]. We see a similar increase of 82% when comparing individuals with two copies of the derived variants (such as GG at rs174537) and two copies of the ancestral variants (TT at rs174537). [57,58]. Figure 1 shows how the dramatic differences in metabolic flux across this rate-limiting step can markedly influence the balance between ARA and other *n*-6 and *n*-3 LC-PUFAs in membrane phospholipids. This in turn determines the levels and ratios of substrates available for phospholipase hydrolysis and *n*-6 and *n*-3 oxylipin biosynthesis.

Given this strong genetic influence on LC-PUFA biosynthesis, we postulate that levels and ratios of *n*-6 to *n*-3 LC-PUFAs interact with the derived haplotype in a large proportion of individuals with African ancestry to produce high (potentially excessive) levels of ARA and its metabolic products. This could also be observed in ~45% of European-ancestry populations. Elevated levels of ARA and its metabolites relative to *n*-6 DGLA and *n*-3 LC-PUFAs and their metabolites would be predicted to give rise to a pro-inflammatory phenotype in individuals consuming high concentrations of LA from the MWD.

In contrast, the levels and ratios of *n*-6 to *n*-3 PUFAs interact with the ancestral haplotype in a large proportion of individuals of Amerind ancestry, such as Mexicans and Native Americans, to produce low (perhaps inadequate) levels of *n*-3 LC-PUFAs and their metabolic products. We have recently observed *n*-3 LC-PUFA deficiency by examining the impact of *FADS* variation in six Hispanic populations in the Multi-Ethnic Study of Atherosclerosis (MESA) cohort [56]. In this study, *FADS* variation accounted for most of the Amerind ancestry effect on HUFAs, and especially the extremely low levels of *n*-3 LC-PUFAs. *FADS* variation was also strongly associated with several metabolic, inflammatory, and anthropomorphic traits including circulating triglycerides, sICAM, E-selectin and waist-to-hip ratio in Amerind ancestry Hispanic populations [57]. Without necessary levels of *n*-3 LC-PUFAs, there is a reduction of the anti-inflammatory, pro-resolution and lipid lowering functions necessary for protection against obesity, CVD, diabetes and non-alcoholic fatty liver disease (NAFLD).

It is also important to mention that *n*-3 PUFA supplementation has been reported to affect *FADS* desaturase activities [61]. After 210 subjects consumed 5 g/day of fish oil for 6 weeks, ∆-5 desaturase activity was increased by 25.7% (*p* < 0.0001) and ∆-6 desaturase was elevated by 39.5% (*p* < 0.0001). These data suggest that *n*-3 PUFA supplementation may affect the enzymatic activities encoded in the *FADS* gene cluster. [61] Further studies are necessary to better understand the mechanism(s) leading to these alterations in activity.

All of this research suggests that a “one size fits all” intervention with *n*-3 LC-PUFA-containing oils is unlikely to be clinically effective across racial/ethnic groups given the dramatic population differences in the frequencies of genetic variants that impact *n*-3 3 LC-PUFA synthesis and levels. The potential impact of these gene by diet interactions is likely very important in individuals with a very high proportion of Amerind ancestry, such as Native Americans, in which case >80% of individuals are likely to have two copies of ancestral *FADS* variants. This is the reverse of African Americans in which >80% of individuals have two copies of the derived-pathway efficient variants. This later group has a much higher conversion of DGLA to ARA while the former has reductions in ARA production. Since they both experience a 10:1 ratio of dietary LA to ALA entering the pathway, the African ancestry population makes high levels of ARA and have some elevation in EPA and DHA compared to other populations. However, we postulate that this increase in *n*-3 3 LC-PUFA does not offset the elevated levels of ARA and its metabolites. In contrast, the 10:1 ratio of LA to ALA together with the ancestral haplotype allows for lower but sufficient synthesis of ARA but extremely low levels of endogenously-formed EPA and DHA [57]. Given the dramatic differences in the endogenous capacity to synthesize *n*-6 and *n*-3 3 LC-PUFA in different individuals and populations, it is not surprising that recent clinical trials and meta-analyses have questioned the efficacy of *n*-3 LC-PUFA supplementation in general populations [22,23,62,63,64,65]. We posit that individual levels of circulating and tissue *n*-3 3 LC-PUFAs produced by the aforementioned gene by diet interactions will directly impact the effectiveness of *n*-3 PUFA supplementation.

## 4. *N*-3 LC-PUFA and Cardiovascular Risk

Cardiovascular disease (CVD) continues to be the primary cause of death worldwide [66] and represents a complex and multifactorial disease process characterized by dyslipidemias, oxidative stress and inflammation that results in atherosclerosis and eventually cardiac death [67]. Dietary strategies are a first line prevention strategy to reduce CVD risk and comorbidities. CVD disproportionately impacts certain racial/ethnic minority populations such as African Americans [68,69]. For example, African Americans have elevated risk for heart disease with the highest age adjusted rates among US populations [70]. African Americans also have elevated rates of hypertension [71] and inflammation [72,73]. 

The Hispanic population is the largest racial/ethnic minority in the US (about 18% of the population) [74]. Hispanics have higher rates of obesity, poorly controlled high blood pressure, elevated circulating triglycerides and a higher prevalence of T2D. Importantly, self-reported Mexican Americans from states bordering Mexico represent two thirds of the US Hispanic population. This population has the highest levels of Amerind ancestry among all US Hispanic populations [75] and are twofold more likely than other racial or ethnic groups, including other Hispanic subgroups, to have cardiometabolic diseases including NAFLD [76]. The rates of T2D, CVD and stroke are higher in Native Americans than in other US populations [77,78]. Chronic liver disease was the fifth leading cause of death (according to the CDC) among Native American and Alaska Natives in 2013 [79]. 

Since pioneering studies by Bang and Dyerberg in Greenland Eskimos in the 1970s, [80,81] *n*-3 LC-PUFAs have been shown to be beneficial in lowering CVD risk in multiple studies by numerous mechanisms including lowering lipids (triglycerides and cholesterol esters) and reducing inflammation [82] leading to reduced cardiac events [18] and deaths [18]. Erythrocyte levels of *n*-3 LC-PUFAs, an established biomarker to determine dietary *n*-3 HUFA exposure are lower in individuals who have experienced a cardiac event or cardiac death [18]. Similarly, a recent study shows higher circulating levels of marine *n*-3 LC-PUFA are associated with lower risk for all-cause mortality, CVD and cancer [83].

Despite earlier promising supplementation studies, more recent studies have questioned the beneficial effects of *n*-3 LC-PUFA, suggesting no clear benefits of *n*-3 LC-PUFAs in cardiovascular or treatment of psychiatric disorders [12,13]. 

For example, a recent meta-analysis of 10 trials from 1999 to 2010 involving 177,917 patients [84,85,86,87,88,89,90] over a period of 4.4 years concluded that **n*-3* LC-PUFAs had no effects on fatal or non-fatal coronary heart disease events [21]. In contrast, Kris-Etherton et al. [28] report that a close evaluation of these trials reveals that in the earlier trials, *n*-3 HUFAs did have an effect on cardiovascular end points [84,85,86] whereas more recent clinical trials reported neutral effects [87,88,89,90]. These authors [28] attribute the discrepancies between trials to the improved standard of care in the more recent studies [85,86,87,88]. Furthermore, they point out that although recent trials ASCEND and VITAL do not find significant differences compared to placebo in their primary end point, the secondary points, including vascular death and risk for heart attack, were improved [28].

There is little information regarding gene–diet interactions between *FADS* and *n*-3 supplementation. A recent report from the Tennessee Polyp study (n = 141 subjects) [91] evaluated the effect of fish oil supplementation and gene variant interactions by stratifying subjects into *FADS* genotypes. They reported that although fish oil supplementation reduced the concentrations of urinary prostaglandin E metabolite, and there was not a significant *FADS* variant diet interaction. It is evident that more studies with a greater number of subjects where the participants are stratified by *FADS* variation are needed to better understand diet-gene interactions. 

Given the genetic and biochemical mechanisms discussed above including the competition between *n*-6 and *n*-3 PUFAs in the 3 LC-PUFA biosynthesis pathway and in the differences in *FADS* haplotype frequencies among populations, it is not surprising that different populations have varying capacities to synthesize circulating and tissue levels of *n*-3 and *n*-6 3 LC-PUFAs. We propose that this is likely is a major factor in the heterogeneity of clinical trials results. For example, European Americans have relatively high proportions of individuals with two, one or no copies of the derived variants associated with enhanced pathway efficiency. Consequently, there are marked differences in the levels of *n*-6 and *n*-3 3 LC-PUFAs in each of these three groups [51,53,92] within European American populations. For these reasons, *n*-3 supplementation likely will not benefit all European American individuals universally. In contrast, >80% of African Americans have two copies of the derived *FADS* variants, and >80% of Native Americans have two copies of ancestral *FADS* variants, all giving rise to marked differences in the capacity to synthesize endogenous *n*-6 and *n*-3 LC-PUFA s. This accounting for endogenous *n*-6 and *n*-3 Lc-PUFAs levels may be why studies that examine associations between circulating or erythrocyte *n*-3 LC-PUFAs and CVD or all-cause mortality have been more consistent than supplementation studies [18]. 

A closer examination of several recent large *n*-3 LC-PUFA supplementation trials supports the gene by diet influence by providing evidence that not only does *n*-3 supplementation benefit some groups, but the groups that appear to benefit most are those groups expected to do so based on genetic variation in the 3 LC-PUFA biosynthetic pathway. For this reason, it is worth revisiting the results from recent large clinical trials: the VITAL trial [22], the ASCEND trial [23] and the REDUCE-IT trial [26,27]. 

The ASCEND study recruited 15,840 individuals with diabetes between 2005 and 2011 who were randomized either to 1 g capsule of **n*-3* HUFAs (0.41 g of EPA and 0.34 g of DHA) or a matching placebo (containing olive oil) [23]. The primary outcome was the first serious cardiovascular event including nonfatal myocardial infarction or stroke, transient ischemic attack or vascular death, with a follow up of 7.4 years. No significant differences were found in the composite primary outcome between *n*-3 3 LC-PUFA and placebo; however, authors reported that for secondary events, there was an 18% decrease in the rate of cardiovascular death when comparing *n*-3 3 LC-PUFA supplementation versus placebo [23] (Table 1).

The REDUCE-IT trial enrolled 8179 patients from 11 different countries including United States, Canada, The Netherlands, Australia, New Zealand, South Africa, Poland, Rumania, Ukraine, Russia and India [26]. The intervention consisted in consuming 2 g of EPA as an ethyl ester twice a day for a total of 4 g versus a placebo. The major findings from this study were that the risk of ischemic events including cardiovascular death were reduced in the EPA group compared to placebo even though all participants were being treated with statins [26]. In addition, there were significant differences in several secondary outcomes. 

The REDUCE-IT USA trial is a subgroup that consists of 3146 patients from the US, of which 32.3% were women and 9.7% were Hispanic [27]. The authors emphasized that the US subgroup demonstrated particularly strong reductions across individual and composite cardiovascular points when compared to the non-US groups. These reductions were observed in cardiovascular death, myocardial infarction, stroke and all-cause mortality (Table 1).

The VITAL Vitamin D and *n*-3 3 LC-PUFA Trial consisted of 25,871 men aged over 50 years old and women over 55, including 5106 African Americans who received a daily dose of 460 mg of EPA plus 380 mg of DHA or placebo [22]. The median intervention lasted 5.3 years. Authors reported no differences between *n*-3 3 LC-PUFA supplementation and placebo for their primary end point of major cardiovascular events; however, the rate of myocardial infarction was significantly reduced by 28%. Furthermore, the information provided in the study’s Supplementary Appendix Materials (Supplementary Table S4 and Supplementary Figure S3) [22] indicate that supplementation of *n*-3 HUFAs resulted in a significant reduction in total mortality in those who consumed less than 1.5 servings (approx. 255 g) per week of fish versus those who consumed >1.5 servings [22] supplementary material. Perhaps the most important information from this study is presented in Supplementary Figure S3 [22] where, after controlling for age, sex and vitamin D randomization, myocardial infarctions were 71% lower in *n*-3 supplementation versus the placebo in African Americans (Table 1). We propose that these effects are seen in African ancestry populations because of the enhanced capacity (due to the derived haplotype variation) to produce ARA and ARA metabolites. Here, we postulate that *n*-3 HUFA supplementation leads to the production of anti-inflammatory, pro-resolving *n*-3 3 LC-PUFA metabolites necessary to offset the pro-inflammatory effects of ARA metabolism in African Americans.

To summarize, although the primary composite endpoints did not always show differences between treatment groups, there is evidence in all four trials of benefits from *n*-3 supplementation, especially for certain secondary outcomes and subgroups. Despite having increased CVD risk, few *n*-3 3 LC-PUFA supplementation trials have included a large number of African American or Hispanic participants, with most studies conducted primarily on subjects of European ancestry. Consistent with the proposed effects of the *FADS*-derived haplotype in African American populations, the VITAL trial showed that *n*-*3* LC-PUFA supplementation markedly lowers the risk of myocardial infarction in the 5106 African Americans enrolled [22]. This type of sub-analysis was not possible in the REDUCE-IT USA trial given that only 4% of the enrolled participants were African Americans [27]. 

## 5. Summary and Future Recommendations

The *FADS*-impacted endogenous capacity of individuals and populations to synthesize *n*-6 and *n*-3 LC-PUFAs likely plays a critical role in how they respond to *n*-3 LC-PUFA s. It may be that supplementation with *n*-3 3 LC-PUFA s balances the over-production of ARA and its metabolites in African ancestry populations and directly addresses *n*-3 LC-PUFA deficiencies in Amerind ancestry populations. Given this, there is a desperate need for more *n*-3 3 LC-PUFA supplementation trials in those underrepresented populations that may most benefit from them. Currently, there is an urgent need to determine if the efficacy of *n*-3 3 LC-PUFA supplementation on reducing cardiovascular events and mortality observed in African Americans in VITAL can be replicated. Furthermore, as participants eating very little fish also benefited, it is likely that participant *n*-3 LC-PUFA dietary exposure has been a major factor in the mixed results of *n*-3 supplementation trials. Finally, we recommend that a one-size-fits-all approach to *n*-3 LC-PUFA supplementation should be replaced by precision advice tailored to an individual’s diet, ancestry and genetics. 

## Figures and Tables

**Table 1:** Recent randomized, placebo-controlled studies of *n*-3 fatty acids (FA) in the prevention of cardiovascular disease *.

Study	Participant Characteristics	Type and Duration of Intervention	Populations	Primary Outcomes:	Other Outcomes
ASCEND [23]	15,840 diabetics	Median of 7.4 Years, 1 g/day of *n*-3 (0. 41 EPA and 0.34 DHA)	97% White	No difference with Placebo in cardiovascular death	7% reduction in the rate of non-fatal myocardial infarction and 20% reduction in vascular death
REDUCE IT [26]	8179, on statin therapy and TG = 135 to 499 and LD L= 41–100 mg/dL	Median 4.9 years, 2 g of icosapentenyl twice a day or placebo	11 Countries Primarily European Ancestry	26% reduction in rate of composite primary cardiovascular endpoints	Rates of individual end points (except death from any cause lowered by ≥20%
REDUCE-IT, USA [27]	3146 patients	Median 4.9 years, 2 g of icosapentenyl twice a day or placebo	32.3 % women, 9 % Hispanic. 4% African American and 2% Asian	31% reduction in rate of composite primary cardiovascular endpoints	Rates of individual end points (except death from any cause lowered by ≥28%
VITAL [22]	29,581. 50 y old (5,106 subset of AA)	Median of 5.3 years 840 mg/day *n*-3 FA	All Participants African Americans (20%) Subjects who ate less than 1.5 portions of fish per week	No differences in rate of combined major cardiovascular events No significant difference 19% reduction in rate	28% reduction in rate of myocardial infarction 71% reduction in myocardial infarction

* The primary composite end point was cardiovascular death, nonfatal myocardial infarction, nonfatal stroke, coronary revascularization or hospitalization for unstable angina.

## Data Availability

Not applicable. This is a review paper.
